# Efficacy of Three Povidone Iodine Formulations against *Cutibacterium acnes* Assessed through In Vitro Studies: A Preliminary Study

**DOI:** 10.3390/antibiotics11050665

**Published:** 2022-05-16

**Authors:** Maxime Pichon, Christophe Burucoa, Victor Evplanov, Filippo Favalli

**Affiliations:** 1 Bacteriology Laboratory Poitiers, Infectious Agents Department, Pharmacology of Antimicrobial Agents and Antibioresistance, Faculté de Médecine et Pharmacie, University Hospital of Poitiers, INSERM U1070, 86034 Poitiers, France; christophe.burucoa@chu-poitiers.fr; 2 Mylan, A Viatris Company, 75008 Paris, France; victor.evplanov@viatris.com; 3 Meda Pharma S.P.A., A Viatris Company, 20900 Monza, Italy; filippo.favalli@viatris.com

**Keywords:** *Cutibacterium acnes*, skin microbiota, antiseptic, microbiology, prosthetic joint infection

## Abstract

*Cutibacterium acnes* (*C. acnes*, formerly known as *Propionibacterium acnes*) is the major causative agent of prosthetic joint infections (PJI). Treatment of PJI with antibiotics is difficult due to antibiotic resistance and adverse side effects on patients’ health. Proper disinfection of the surgical site using a variety of povidone iodine formulations could prevent *C. acnes* infection. In the current study, the efficacy of the three povidone iodine (PVP-I) formulations, viz: PVP-I 10% dermic solution, PVP-I 5% alcoholic solution and PVP-I 4% scrub, was tested against *C. acnes*, in vitro, in the presence of interfering substances mimicking soiling conditions. *C. acnes* strain ATCC 6919 was used to test the bactericidal activity of the povidone iodine formulations according to the modified dilution-neutralization method described in French Norm EN standard 13727. A 3-log reduction in the bacterial cell count in 60 s was considered to be significant. The results showed that under experimental conditions, the three PVP-I formulations displayed bactericidal activity against the micro-organism, *Cutibacterium acnes,* and that the lowest concentration of povidone-iodine active against *C. acnes* was 0.4%. These results are encouraging as PVP-I offers a low-cost and efficient method of disinfection.

## 1. Introduction

*Cutibacterium acnes* (*C. acnes*, formerly known as *Propionibacterium acnes*) is an anaerobic, gram-positive bacterium that is a major cause of prosthetic joint infection (PJI) after shoulder arthroplasty [1]. *C. acnes* is mainly found in the dermal and epidermal layers of the skin and often causes contamination of surgical instruments [2]. Recent studies have shown that *C. acnes* is involved in most post-operative shoulder infections [3,4].

Six main phylotypes of *C. acnes* have been identified to date, viz. IA_1_, IA_2_, IB, IC, II and III, of which types IB, II and III are the main causes of PJI [5]. *C. acnes* is also known to form biofilms having a high virulence and increased antibiotic resistance in prosthetic implants [6]. Antibiotics like beta-lactams, quinolone, rifampicin, and clindamycin are generally used for the treatment of PJI. The treatment period is typically three months, but there is an increased risk of failure due to antibiotic resistance, late prognosis, and adverse side effects on a patient’s health due to the use of antibiotics [4]. Hence, proper disinfection of the skin is required to prevent *C. acnes* infections.

Skin preparation is carried out prior to any surgical procedure. It generally involves the application of antiseptics to the skin surface using a sterile compress for about 10 s and then allowing it to dry for around 30 s [7]. Alcohol-based skin disinfectants have been shown to be ineffective against *C. acnes* [8]. Other antiseptic agents used for skin preparation are chlorhexidine gluconate (CHG) and hydrogen peroxide. The moderate efficacy of hydrogen peroxide as a disinfectant for *C. acnes* was shown in a non-randomized, single blind trial with 124 subjects [9]. A prospective study in 100 patients with 2% CHG did not show any significant reduction in *C. acnes* colonization compared to the control [10].

Another widely used disinfectant is povidone iodine (PVP-I). It is a water-soluble complex that can be considered for the disinfection of *C. acnes* thanks to its immediate onset of action, greater skin permeation, broad antimicrobial spectrum, lack of resistance, efficacy against biofilms and good tolerability [11]. The Centers for Disease Control and Prevention (CDC) and the World Health Organization (WHO) recommend the use of dilute povidone-iodine solution prior to wound closure [12,13,14]. A substantial benefit of alcohol-based povidone-iodine compared to alcohol-based chlorhexidine was shown in controlling the anaerobic microflora of the skin in a study with 16 volunteers [15].

PVP-I is available in a variety of formulations [16]. PVP-I 10% dermic solution is used as an antiseptic for wounds or superficial burns covering a small surface area, for the local adjunct treatment of skin disorders and infected mucosa or when there is a risk of infection, and for skin preparation of the surgical field [17]. This formulation (Povidone iodine 10 g per 100 mL, Glycerol, Macrogol lauric ether, Disodium phosphate dihydrate, Citric acid monohydrate, Sodium hydroxide, Purified water) can be used for washing wounds, pure (by brushing on the skin) or diluted to a tenth (with water or saline); and for wound irrigation (diluted to a fifth with saline). PVP-I 5% alcoholic solution is applied as an antiseptic to healthy skin prior to surgery or before certain invasive procedures [18]. It is recommended that one apply five milliliters of this formulation (Povidone iodine 5 g per 100 mL, 96% ethanol 72 mL per 100 mL, glycerol, macrogol lauric ether, purified water for an alcohol content of 69.2% *v/v*) on the skin with a sterile compress within 10 s (before 30 s of drying). PVP-I 4% scrub is recommended for cleansing and as an adjunct treatment in skin and mucosal infections that are primarily bacterial or likely to become superinfected; for antiseptic washing of the hands of the nursing staff and the hands of the surgeon; and as a preoperative antiseptic wash [19]. This formulation (Povidone iodine 4 g per 100 mL, 60% ammonium salt solution of alkylphenoxypolyethylene ethane sulfuric ester, bishydroxyethyllauramide, sodium hydroxide, purified water) can be used for the preparation of the patient (pure), for the washing of soiled wounds (product diluted to one third with water or physiological serum); and for the hygienic washing of the hands (4 mL of pure solution on wet hands). In the current study, the efficacy of the three aforementioned PVP-I formulations was tested against *C. acnes*, in vitro, in the presence of interfering substances mimicking soiling conditions.

## 2. Results

All three formulations displayed a bactericidal activity against the micro-organism. As detailed in the methods section of the manuscript and in the respective part of the results section, all controls allowed for the validation of the process, since the numbers of bacterial CFU in controls A, B and C were evaluated to be more than 0.5 times the Nv (ratio indicated in square brackets in the respective result paragraphs).

*C. acnes* ATCC 6919 with the hygienic hand wash was determined according to an adaptation of the protocol proposed in french standard EN 13727 and the amended dilution-neutralization method for ready-to-use products under conditions of soiling at 20 °C in 60 s.

### 2.1. PVP-I 4% Scrub

The initial cell count of the test suspension was 1.65 × 10^9^ cells (N_0_). The cell count after contact time was 140. A log reduction of >7.06 was observed for both undiluted and 10^−1^ dilutions. At a 10^−2^ dilution, the log reduction was 3.99 (Table 1). The colony count for the validation suspension was 1031 CFU/mL. The colony counts for controls A, B and C were 650 CFU/mL [0.63], 1339 CFU/mL [1.30], and 656 CFU/mL [0.64], respectively.

### 2.2. PVP-I 5% Alcoholic Solution

The initial cell count of the test suspension was 1.65 × 10^9^ cells (N_0_). After contact time, the cell count was 140. A log reduction of >7.06 was also observed for 5% PVP-I, like for 4% PVP-I scrub. At dilutions of 10^−1^ and 10^−2^, the log reduction was >5.9 and 4.6, respectively (Table 1). The colony count for the validation suspension was 38 CFU/mL. The colony counts for controls A, B and C were 45 CFU/mL [1.18], 115 CFU/mL [3.03], and 43 CFU/mL [1.13], respectively.

### 2.3. PVP-I 10% Dermic Solution

The initial cell count of the test suspension was 1.65 × 10^9^ cells (N_0_). After contact time, the cell count was 140. A log reduction of >7.06 was also observed for 5% PVP-I, like for 4% PVP-I scrub. At dilutions of 10^−1^ and 10^−2^, the log reduction was >5.9 (Table 1). The colony count for the validation suspension was 38 CFU/mL. The colony counts for controls A, B and C were 45 CFU/mL [1.19], 115 CFU/mL [3.03], and 43 CFU/mL [1.13], respectively.

## 3. Discussion

*C. acnes* is a human skin flora often implicated in orthopedic infections. The unique characteristics of this microorganism make the diagnosis of an infection difficult [20]. Antibiotic prophylaxis measures are particularly important, but they are not efficient since infection may still occur due to the antibiotic resistance of the microbe [21]. Povidone-iodine is a chemical complex of polyvinylpyrrolidone and elemental iodine. Free iodine is gradually released from this complex, which causes its bactericidal effect [22]. It is effective against a wide spectrum of microorganisms including antibiotic-resistant microbes. PVP-I has high bactericidal effects against gram-positive and gram-negative bacteria, fungi, and viruses [13]. A study has shown that prior to surgical procedures, rinsing the skin with 3.24% alcoholic PVP-I solution is an effective method for reducing SSI and PJI caused by *C. acnes* [15].

The current study tested the in vitro bactericidal activity of three PVP-I formulations: PVP-I 4% scrub, PVP-I 5% alcoholic solution and PVP-I 10% dermic solution. A 3-log reduction in the bacterial cell count in one minute maximum, under “dirty” conditions, was considered as a significant bactericidal activity of the formulations (EN 13727 + A2). This study showed that all three formulations have a bactericidal activity against this pathogen. The colony counts in the validation controls A, B and C for each product were >0.5 N_V_, suggesting that the adopted method was justified. Undiluted concentrations of PVP-I 10%, PVP-I 5% and PVP-I 4% showed an equal efficacy in reducing the *C. acnes* cell count within 60 s contact time. In the case of a ten-fold dilution, PVP-I 4% scrub caused the highest reduction in the *C. acnes* cell count.

Due to the *C. acnes* localization (inside of skin follicles), skin cleaning prior to the application of the antiseptics could probably facilitate antiseptic penetration inside follicles. Cleaning skin with soap alters its properties, leading to a better permeation [23]. Studies with 0.75% PVP-I scrub have shown its capability to eliminate *C. acnes*-related infections in patients having undergone shoulder surgery [24]. In the present study, 0.40% PVP-I scrub showed a greater efficacy compared to the other two formulations, as it cleaned the skin pores, leading to a better permeability of the drug (Table 1). Recent in vitro studies have shown that a 0.35% PVP-I solution (in NaCl) is effective in controlling *C. acnes* infection, which is similar to the results in the present study [25]. A retrospective study in 1862 patients having undergone arthroplasty showed a significant reduction in surgical site infection (0.97% to 0.15%, *p* = 0.04) when 0.35% PVP-I was used for wound closure [26]. In non-PJI infections, in both in vitro and in vivo evaluations, the 0.6% ophthalmic formulation of PVP-I was more rapidly bactericidal than the 5% formulation on a variety of clinical and non-clinical *staphylococci* (including *S. aureus*), gram-negative bacilli (including *Pseudomonas aeruginosa* and *Escherichia coli*) and fungi (*Candida* sp.) [27,28,29]. This difference in efficacy (only present on bacterial strains) is, according to the authors, most likely due to the fact that dilution from 5% to 0.6% increases the amount of free iodine. These studies, even if they do not give data on *C. acnes*, agree with the present results, as the selected strain is also involved in ophthalmic diseases, which could benefit from the use of this antiseptic molecule [5].

The three antiseptic solutions tested (PVP-I 10% dermic solution, PVP-I 5% alcoholic solution and PVP-I 4% scrub) contained the same active substance (povidone-iodine), at different concentrations. The reductions in the bacterial cell count obtained for the three formulations (Table 1) suggest that, under experimental conditions, povidone-iodine is active against *C. acnes* up to a concentration of 0.4%. It is noted that the conditions and validation mixtures of the method do not always generate values within the limits defined in the standard. These results are encouraging as PVP-I offers a low-cost and efficient method of disinfection. Aqueous and alcoholic PVP-I formulations are active for 12 to 14 h and have a good skin tolerance. It was also observed that PVP-I scrub had a better skin tolerance compared to similar antiseptic scrub formulations containing chlorhexidine, benzalkonium chloride or cetrimide [30,31]. Nevertheless, in vitro analyses of the bactericidal efficacy of PVP-I on other pathogens frequently involved in PJI (*Staphylococcus aureus*, coagulase-negative *Staphylococci*, *Streptococci*, etc.) must be carried out to apprehend the overall efficacy of the antiseptic procedure when using this molecule in orthopedic surgery, for example.

## 4. Materials and Methods

### 4.1. Microorganisms and Culture Conditions

*C.acnes* strain ATCC 6919 (ref. CIP53.117T batch 1515-2d; CNR ref. 2019/00808) was supplied by the Pasteur Institute, France. Note that the entire process (including bacterial strains, identification, and growth methods) was validated (data not shown) by the French national reference center for botulism and anaerobic bacteria, which performed the analyses to fit the NF13727 + A2:2015 standard. Briefly, the bacteria were cultured in-house using TGYH medium (composition in (g/L): Trypticase peptone (30), Yeast extract (20), D-glucose (5), L-Cysteine-HCl (0.5) and 25 mL Hemin solution at 37 °C under anaerobic conditions (90% N_2_/5% H_2_/5% CO_2_) in a transparent anaerobic jar (MART AJ 9023) for four to five days. Anaerobiosis was created and maintained in the jars by the Anoxomat Mark II system, which injects a three-gas mixture (90% N_2_/5% H_2_/5% CO_2_). To create a solid culture, 1.6% agar was added to the TGYH medium. The pH of the medium was adjusted to 7.4 and autoclaved at 110 °C and 27 psi (1861 bar) for 30 min for sterilization. Deionized water was used for the medium preparation and experiments throughout the study, unless otherwise stated.

### 4.2. Antiseptic Solution Preparations

PVP-I (Mylan, Canonsburg, PA, USA) 10% dermic solution (Batch 324083: 11/2021 and Batch 324624: 04/2022), PVP-I 5% alcoholic solution (Batch 324183: 11/2022 and Batch 324576: 03/2023) and PVP-I 4% scrub (Batch 324178: 11/2022 and Batch 324742: 05/2023) come as ready-to-use products. For experimental purposes, three concentrations of each product were used, i.e., undiluted, 1:10 dilution and 1:100 dilution. Dilutions were made using sterile injection water (B. Braun, Melsungen, Germany). Dilutions were prepared freshly and used within two hours of preparation.

### 4.3. Experimental Procedure

Bactericidal activity was tested according to the modified dilution-neutralization method described in EN standard 13,727. *C. acnes* was grown in TGYH agar medium for 18 to 24 h. Colonies of *C. acnes* were picked using a sterile loop and suspended in sterile water to form a uniform suspension. This was labelled as a test suspension and was maintained at 20 °C in a water bath. An aliquot (0.2 mL) of test suspension was added to 0.1 mL of interfering substances (3.0 g/L of bovine albumin solution plus 3.0 mL of red blood cells), mimicking soiling conditions and hygienic hand washing. The sample was immediately incubated in a water bath at 20 °C for 2 min. At the end of this time, 9.7 mL of the product test solution were added, and the mixture was maintained at a temperature of 20 °C for 60 s (contact time). After 60 s, a 1 mL sample was collected, and the bactericidal/bacteriostatic activity was neutralized immediately with an NPDT (Neutralising Pharmacopoeia Diluent + Thiosulphate) buffer (8 mL) using the dilution-neutralization method, and 1 mL sterile water was added. The composition of NPDT buffer (pH 7.0) is (g/L): polysorbate 80 (30), egg lecithin (3), histidine HCl (1), peptone from casein (1), NaCl (4.3), KH_2_PO_4_ (3.6), K_2_HPO_4_ (7.2), Na_2_SO_3_ (5). The following bacterial CFU (colony forming unit) count was conducted in each sample by plating on TGYH agar medium, and the reduction rate was calculated according to the following formula:

(1) $$\mathrm{Reduction}=\log N_{0\;}-\log N_{A}$$

where N_0_ is the initial bacterial count in the test suspension and N_A_ is the bacterial count after contact with the antiseptic. N_0_ was calculated for each experimental set (PVP-I 4% scrub, PVP-I 5% alcoholic solution and PVP-I 10% dermic solution).

### 4.4. Validation of the Results and Experimental Protocol

Experimental control A was run to check the absence of bactericidal activity in the neutralizing buffer (NPDT). Control B was run to check the effectiveness of the neutralizing buffer in stopping the bactericidal effect of the product. Control C was run to validate the method of dilution-neutralization. The test suspension was diluted using sterile water to make the validation suspension NV (3.0 × 10^2^ CFU/mL to 1.6 × 10^3^ CFU/mL) and neutralizer control NVB (3.0 × 10^4^ CFU/mL to 1.6 × 10^5^ CFU/mL).

An interfering substance was added to the validation suspension, and the bacterial cell count was done by plating on TGYH medium (control A). To validate the neutralization method (control B), the interfering substance, NPDT buffer, product test solution and neutralizer control were added, and the bacterial cell count was done using the spread plate technique. To validate the dilution neutralization method (control C), the interfering substance was added along with the product test solution, NPDT buffer and validation suspension. The bacterial cell count was done using the spread plate technique.

The process was validated if the bacterial CFU counts in controls A, B and C were >0.5 N_V_. All experimental sets were run in triplicates (*n* = 3) in different batches.

## 5. Conclusions

PVP-I 10% dermic solution, PVP-I 5% alcoholic solution and PVP-I 4% scrub displayed bactericidal activity against the micro-organism *Cutibacterium acnes* ATCC 6919 under conditions of soiling at 20 °C in 60 s. Under the experimental conditions, the products were sufficiently effective to be used for surgical procedures. The lowest concentration of povidone-iodine active against *C. acnes* was found to be 0.4%.

## Figures and Tables

**Table 1:** Log reduction of *Cutibacterium acnes* when treated with different betadine formulations.

Product Name	PVP-I 10% Dermic Solution		PVP-I 5% Alcoholic Solution		PVP-I 4% Scrub	
Product dilution	Actual povidone-iodine concentration	Reduction (log)	Actual povidone-iodine concentration	Reduction (log)	Actual povidone-iodine concentration	Reduction (log)
Undiluted	9.7%	>7.06	4.9%	>7.06	3.9%	>7.06
1/10	0.97%	>5.9	0.49%	>5.9	0.39%	>7.06
1/100	0.097%	>5.9	0.049%	4.66	0.039%	3.99

## Data Availability

Not applicable.
